# Spastic Quadriplegia Resulting From a Pathogenic Variant in the *SPAST* Gene: A First Report

**DOI:** 10.1155/crnm/2632593

**Published:** 2026-04-29

**Authors:** Eirini Kostopoulou, Maria Lagadinou, Ageliki Karatza, Xenophon Sinopidis, Rin Khang, Gabriel Dimitriou

**Affiliations:** ^1^ Department of Paediatrics, University General Hospital of Patras, Patras, 26500, Greece, pgnp.gr; ^2^ Medical School of Patras, University of Patras, Patras, 26504, Greece, upatras.gr; ^3^ Internal Medicine Department, University General Hospital of Patras, Patras, 26500, Greece, pgnp.gr; ^4^ 3billion Inc., Seoul, South Korea

**Keywords:** case report, developmental delay, epilepsy, quadriplegia, SPAST

## Abstract

**Background:**

The *SPAST* gene encodes spastin, a microtubule‐severing protein. Pathogenic variants in this gene are commonly associated with autosomal dominant Spastic paraplegia type 4 (*SPG4*), a neurodegenerative disorder presenting with progressive lower limb spasticity. Severe phenotypes involving more extensive neurological impairment are rare.

**Case Presentation:**

We report the first known case of an adolescent with a rare pathogenic *SPAST* variant (NM_014946.4: c.1507C > T; NP_055761.2: p. [Arg503Trp]) presenting with spastic quadriplegia. Additional features included severe intellectual disability, absent speech, dysphagia, and epilepsy, manifestations infrequently reported in association with *SPAST* mutations. He was born prematurely at 32 weeks of gestation after a complicated antenatal period due to the development of preeclampsia at 23 weeks and gestational diabetes mellitus at 26 weeks.

**Discussion:**

The patient’s clinical presentation represents one of the most severe phenotypes described in the literature for *SPAST*‐related disorders. The identification of a genetic cause provided a more satisfactory and specific diagnosis than the previously presumed etiology of perinatal asphyxia. This case broadens the phenotypic spectrum of *SPAST*‐related disease and highlights the role of genetic testing in the diagnostic workup of complex neurological conditions.

**Conclusion:**

We report the first case of a pathogenic variant in the *SPAST* gene and quadriplegia and one of the most severe clinical phenotypes described in the literature in association with variants in this gene. This report underscores the importance of considering *SPAST* mutations in patients with atypical or severe neurodevelopmental presentations. Genetic diagnosis enables more accurate prognosis, individualized medical care, and appropriate genetic counseling for families.

## 1. Introduction

The *SPAST* gene, previously known as the Spastic paraplegia type 4 (*SPG4*) gene, encodes the microtubule‐severing protein called spastin, a member of the ATPases associated with diverse cellular activities (AAA) protein family, and is associated with autosomal dominant *SPG4* (OMIM: #182601). *SPG4* is a neurodegenerative disorder presenting with progressive stiffness and weakness of the lower extremities and accounts for up to 40%–45% of familial cases [1–3]. Nearly 75% of the *SPG4* cases are inherited [4].

The typical presentation of *SPG4* includes isolated lower limb spasticity, hyperreflexia, and slowly progressive gait disturbance, usually emerging between the second and fourth decades of life. However, significant phenotypic variability has been reported, even within families. Complex forms of *SPG4* may include additional features such as upper limb spasticity, cognitive impairment, epilepsy, ataxia, and dysarthria [5, 6]. Despite this variability, the occurrence of severe neurodevelopmental delay and profound intellectual disability in association with *SPAST* variants remains exceedingly rare. Interestingly, patients carrying *de novo* variants in the *SPAST* gene have been reported to exhibit more complex phenotypes with progressive cognitive dysfunction and intellectual disability, as well as onset at a younger age compared to patients with familial SPG carrying the same variants [4].

In this report, we describe a unique case of an adolescent with a known rare pathogenic *SPAST* variant (NM_014946.4: c.1507C > T; NP_055761.2:p. [Arg503Trp]), presenting with a highly atypical and severe clinical phenotype characterized by spastic quadriplegia, severe intellectual disability, absent speech, epilepsy, and swallowing difficulties. The case expands the known phenotypic spectrum of *SPG4* and underscores the importance of considering a genetic diagnosis in patients with complex neurological presentations.

## 2. Case Presentation

We present the case of a 14‐year‐old male with a complex neurodevelopmental profile characterized by spastic quadriplegia, global developmental delay, and a history of West syndrome evolving into refractory epilepsy. There was no relevant family history. The patient was conceived via in vitro fertilization (IVF) and was born prematurely at 32 weeks of gestation following a high‐risk pregnancy. The antenatal period was complicated by the development of preeclampsia at 23 weeks and gestational diabetes mellitus at 26 weeks, the latter managed with insulin therapy. Fetal monitoring later revealed pathological findings on nonstress testing (NST), prompting an emergency cesarean section.

At birth, the infant’s anthropometric measurements included a birth weight of 2040 g, a length of 49 cm, and a head circumference of 32 cm, placing him in the appropriate range for gestational age. Soon after delivery, he exhibited signs of mild respiratory distress and was managed initially with an oxygen hood. Within the first 24 h, his respiratory status deteriorated, necessitating endotracheal intubation and mechanical ventilation for 48 h. The neonatal course was further complicated by prolonged hospitalization in the neonatal intensive care unit (NICU), during which he demonstrated hypotonia, poor feeding, and intermittent apnea, raising early concerns for underlying neurological impairment.

At the age of 8 months, the patient was diagnosed with West syndrome, presenting with clusters of infantile spasms, a markedly disorganized electroencephalographic pattern consistent with hypsarrhythmia, and evidence of mild motor delay. Initial treatment with vigabatrin yielded excellent seizure control and was maintained for 6 months; subsequently, the patient transitioned to valproic acid as maintenance therapy.

Between the ages of 1 and 3 years, the patient experienced three episodes of complex focal seizures, two of which escalated to status epilepticus, requiring emergency intervention and hospitalization. Seizure control was eventually achieved through a multidrug regimen consisting of valproic acid, levetiracetam, and topiramate.

Neuroimaging with brain MRI revealed structural abnormalities, including a thin corpus callosum and diffuse cerebral atrophy. A video EEG performed at 3 years of age demonstrated diffuse encephalopathic activity.

Based on the clinical presentation, seizure history, neuroimaging, and EEG findings, early infantile epileptic encephalopathy was diagnosed, hypothetically secondary to perinatal asphyxia in the context of prematurity and neonatal complications.

Following the onset of West syndrome at 8 months of age, the patient’s clinical course was marked by progressive neurodevelopmental decline. He developed secondary microcephaly, with head circumference falling progressively below the third percentile. Over time, a spastic quadriplegic motor pattern became evident, more pronounced on the right side, accompanied by progressive muscle wasting and joint contractures. Severe intellectual disability and global developmental delay, characterized by a developmental quotient of around 20, were also noted, with the patient failing to attain meaningful milestones in language, motor, and adaptive functioning.

Neurological examination revealed reduced muscle strength (Grade 1 of the Medical Research Council Scale on the right side and Grade 4 on the left side), central hypotonia, and increased muscle tone, particularly on the right side (spasticity). Reduced motor coordination, increased tendon reflexes, and unaffected pain sensation were also identified. All abovementioned clinical milestones are presented in chronological order in Table 1.

**TABLE 1:** Timeline of key clinical milestones: all clinical events are presented in chronological order.

Age	Clinical event
Conception/pregnancy	Conceived via IVF; preeclampsia (23 weeks) and gestational diabetes (26 weeks)
Birth (32 weeks)	Premature birth; weight 2040 g, length 49 cm, head circumference 32 cm; mild respiratory distress requiring oxygen, later intubation and ventilation
Neonatal period	Prolonged NICU stay with hypotonia, poor feeding, and intermittent apnea
8 months	Diagnosis of West syndrome: infantile spasms, hypsarrhythmia on EEG, and mild motor delay. Initiation of treatment with vigabatrin
1–3 years	Multiple complex focal seizures including status epilepticus episodes. Administration of multidrug antiepileptic therapy (valproic acid, levetiracetam, topiramate)
3 years	Thin corpus callosum and cerebral atrophy on brain MRI. Video EEG shows diffuse encephalopathic activity. Diagnosis: early infantile epileptic encephalopathy, likely secondary to perinatal asphyxia
Progression (infancy to childhood)	Development of secondary microcephaly, spastic quadriplegia (more severe on the right side), muscle wasting, joint contractures, severe intellectual disability, global developmental delay
Current (14 years)	Seizure‐free for 11 years on valproic acid and topiramate treatment; profound functional impairment, including nonverbal status, and oropharyngeal dysphagia requiring feeding assistance. Reduced growth parameters (weight, height, and head circumference < 3rd percentile)
Recent genetic testing	Whole exome sequencing identified a pathogenic heterozygous variant in the *SPAST* gene (Arg503Trp)

An extensive metabolic workup, including serum amino acids, urine organic acids, plasma lactate and pyruvate, ammonia, and acylcarnitine profile, was conducted and yielded normal results, effectively ruling out an underlying inborn error of metabolism as the cause of his neurological condition.

Whole exome sequencing (WES) was recently performed to exclude a genetic etiology. Analysis revealed a heterozygous pathogenic variant in the *SPAST* gene, located on Chromosome 2p22.3. The identified variant, designated as NC_000002.11: g.32366986C > T (NM_014946.4:c.1507C > T; NP_055761.2:p. [Arg503Trp]), results in a missense substitution of arginine to tryptophan at Codon 503, a residue highly conserved across species. The variant’s nomenclature is based on HGVS and EJHG guidelines (https://www.nature.com/ejhg/authors-and-referees/policies), verified in VariantValidator (https://variantvalidator.org/). Variant 1 was detected in our patient (Table 2). The same nucleotide change resulting in the same amino acid change and different missense changes at the same codon (NM_014946.4: c.1508G > T, NP_055761.2: p. [Arg503Leu], NM_014946.4: c.1508G > C, NP_055761.2: p. [Arg503Pro]) have been reported to be associated with *SPAST*‐related disorder [3]. According to the American College of Medical Genetics and Genomics (ACMG) guideline for genetic variant classification, variants are classified as pathogenic, likely pathogenic, uncertain significance, likely benign, or benign. The classification is based on the functional impact according to a combination of population, computational, functional, and segregation data [7]. Specifically, benign variants are those with a low functional impact based on published data (probably benign variants) or predicted to be benign by in silico tools (predicted benign variants). Pathogenic variants are those with a high functional impact based on published data or predicted to be pathogenic by in silico tools. Variants of unknown significance (VUS) are variants that do not fit into the two previous categories.

**TABLE 2:** Variants in the *SPAST* gene that are described in the literature.

Variant 1	Variant 2	Variant 3
Gene *SPAST*	Transcript NM_014946.4:c.1508G > T	Transcript NM_014946.4: c.1508G > C
Genomic position (GRCh37) NC_000002.11:g.32366986C > T	Protein NP_055761.2:p.(Arg503Leu)	Protein NP_055761.2: p.(Arg503Pro)
Transcript NM_014946.4:c.1507C > T		
Protein NP_055761.2:p.(Arg503Trp)		

No genetic abnormality was detected in any of the first‐degree relatives.

Currently, at 14 years of age, he has been seizure‐free for the past 11 years under treatment with a combination of valproic acid and topiramate. He is also on ongoing neurohabilitation, including speech therapy, physiotherapy, and occupational therapy. Despite sustained seizure control, he presents with profound functional impairment, including absent expressive speech and oropharyngeal dysphagia, requiring dietary modifications and feeding assistance. No dysmorphic features are noted on physical examination, and growth parameters (weight, height, and head circumference) are reduced (< 3^rd^ centile).

The study was in accordance with the ethical standards as laid down in the 1964 Declaration of Helsinki and its later amendments, and it was approved by the Research Ethics Committee of the University General Hospital of Patras (approval number: 64904). Written informed consent was obtained from the patient’s parents for publication of his details.

## 3. Discussion

Hereditary spastic paraplegias (HSPs) are inherited disorders with a broad clinical and genetic spectrum and both interfamilial and intrafamilial variation. They are characterized by progressive lower extremity spasticity and weakness. A locus on Chromosome 2p was first identified in 5 French and 1 Dutch families with autosomal dominant familial spastic paraplegia, and the mean age at onset was 20–39 years [8].

Durr et al. also described 12 families with autosomal dominant spastic paraplegia and the same locus on Chromosome 2p. Affected patients were between infancy and 63 years old, and there was extensive intra‐ and interfamilial clinical variation, with the clinical phenotype ranging from asymptomatic patients or mildly affected individuals with spastic gait to severely affected patients who were wheelchair bound [9].

Nielsen et al. reported 5 families with 2p‐linked spastic paraplegia that presented with either nonprogressive congenital spastic paraplegia or adult‐onset progressive spastic paraplegia. Low backache was also present in 47% of the family members [10].

Nance et al. also described 4 families with spastic paraplegia and Chromosome 2 linkage. The age at onset was 5–61 years. The clinical features included mild neurologic signs in some subjects, scissoring gait in all clearly affected patients and increased tendon reflexes, increased muscle tone, increased muscle spasms or leg cramps, leg weakness, or neurogenic bladder in some patients [11].

### 3.1. Novelty of Quadriplegia

Of particular interest, the presented patient has severe spastic quadriplegia, not paraplegia, accompanied by severe intellectual disability and epilepsy during the first years of life. To our knowledge, this is the first reported case of quadriplegia associated with a pathogenic *SPAST* variant, representing one of the most severe phenotypes described in the literature, presumably related to this gene.

Among the other clinical characteristics of the patient, cognitive impairment and intellectual disability have been described in association with variations in the *SPAST* gene. Specifically, cognitive impairment presenting as deficits in visual–spatial functions and forgetfulness has been reported in an Irish family with autosomal dominant HSP [12]. Reid et al. also showed a significant reduction in scores of the Mini‐Mental State Examination in individuals of Welsh origin with HSP and linkage to the Chromosome 2 locus. Subtle changes in cognitive function that may begin after 40 years of age and be more pronounced after 60 years have also been reported [13, 14], as well as dementia in one patient with *SPG4* [15]. Intellectual disability has also been described in 2 patients from Scotland and 6 patients from Italy [16, 17].

In patients with early onset of clinical manifestations, spasticity of lower extremities, delayed milestones, and cognitive impairment were frequently documented, in contrast to the less common occurrences of speech disorders, epilepsy, and cerebellar or optic nerve atrophy [18–24]. Of note, there are no reported cases of early or late onset of clinical symptoms carrying variants in the *SPAST* gene that presented with quadriplegia, and this is the predominant difference between our case report and existing literature. The co‐occurrence of multiple severe additional symptoms in the same patient makes our case even rarer.

### 3.2. Rare Clinical Features

Furthermore, other clinical features that were present in our patient, such as swallowing difficulties, have also been described in patients with progressive walking difficulties resulting in wheelchair dependence, stiffness of the upper limbs, bladder dysfunction, and dysarthria [25]. Also, epilepsy, which was present in our case from early life, has previously been associated with *SPAST* variants in patients presenting with lower limb spasticity, hyperreflexia, pes cavus, and distal muscle wasting [26].

### 3.3. *SPAST* Variant and Potential Consequences of Arginine to Tryptophane Substitution

The variant of the presented patient is extremely rare, with an allele frequency of less than 0.001% in the Genome Aggregation Database (gnomAD V2.1.1). It is situated within a known mutational hotspot of the *SPAST* gene, which also includes several established pathogenic variants (see Table 2). *In silico* predictive algorithms support a deleterious impact of the variant on the gene or the gene product (missense change): the REVEL score is 0.92, and the 3Cnet score is 0.99, both of which are strongly suggestive of pathogenicity. The same nucleotide change resulting in the same amino acid change has been previously reported as pathogenic/likely pathogenic with strong evidence (ClinVar ID: VCV000219575).

The genetic finding provides molecular confirmation of a HSP 4 phenotype [2] inherited in an autosomal dominant manner, although the clinical presentation in this patient is atypically severe. It is, however, possible that the patient’s clinical severity is compounded by his perinatal complications. This genetic finding could offer a more satisfactory explanation for the clinical phenotype of the patient, including the deceleration in head circumference growth and the encephalopathic findings, compared to the initially presumed, but never actually substantiated, perinatal asphyxia, particularly in the absence of severe respiratory distress soon after birth. Perinatal asphyxia was hypothesized due to the abnormal NST prior to the emergency cesarean section and in the initial absence of alternative potential causes of the patient’s neurological status. Also, the possibilities of nonpaternity or germinal mosaicism were not investigated, which represents a limitation for the conclusions.

## 4. Conclusion

We describe a very rare case of a patient with a variant in the *SPAST* gene. This is the first reported case of a pathogenic variant in the *SPAST* gene and quadriplegia, and, hypothetically, one of the most severe clinical phenotypes described in the literature in association with variants in this gene. Interestingly, the identification of a possible genetic etiology for the patient’s symptoms has provided a different viewpoint for his family and physicians. Furthermore, the presentation of this rare case contributes to our evolving understanding of the *SPAST* gene and emphasizes the significance of WES and genetic testing in cerebral palsy‐like phenotypes. Increased awareness and early diagnosis of such rare and severe clinical entities can facilitate the provision of optimal medical care and genetic counseling.

## Author Contributions

E.K. contributed to data extraction and curation and wrote the first draft. E.K. and G.D. conceived and designed the work that led to the submission. R.K. performed the genetic analysis. M.L., A.K., and X.S. contributed to data extraction. E.K. wrote the first draft of the manuscript. M.L., A.K., X.S., R.K., and G.D. revised the manuscript.

## Funding

The publication of this article in OA mode was financially supported by HEAL‐Link.

## Disclosure

All authors approved the final version of the article and agreed to be accountable for all aspects of the work in ensuring that questions related to the accuracy or integrity of any part of the work are appropriately investigated and resolved.

## Conflicts of Interest

E.K. was granted free‐of‐charge genetic testing (WES) by 3billion. The remaining authors declare no conflicts of interest.

## Data Availability

The data that support the findings of this study are available from the corresponding author upon reasonable request.
